# Social participation needs of older adults living in a rural regional county municipality: toward reducing situations of isolation and vulnerability

**DOI:** 10.1186/s12877-020-01849-5

**Published:** 2020-11-07

**Authors:** Mélanie Levasseur, Sonia Routhier, Irma Clapperton, Chantal Doré, Frances Gallagher

**Affiliations:** 1 School of Rehabilitation, Faculty of Medicine and Health Sciences, University of Sherbrooke, 3001, 12e Avenue Nord, Sherbrooke, Québec J1H 5N4 Canada; 2 Research Centre on Aging, Estrie Integrated University Health and Social Services Centre – Sherbrooke University Hospital Center (CIUSSS de l’Estrie – CHUS), Sherbrooke, Québec Canada; 3 Public Health Direction, CIUSSS de l’Estrie – CHUS, Sherbrooke, Québec Canada; 4 School of Nursing Sciences, Faculty of Medicine and Health Sciences, University of Sherbrooke, Sherbrooke, Quebec Canada; 5 University Institute for Primary Health Care & Social Services, CIUSSS de l’Estrie – CHUS, Sherbrooke, Québec Canada; 6 Research Centre, CIUSSS de l’Estrie – CHUS, Sherbrooke, Québec Canada

**Keywords:** Aged, Community-based participatory research, Social participation, Social planning, Rural population

## Abstract

**Background:**

Social participation is restricted for approximately half the older adult population but is critical in fostering community vitality, promoting health, and preventing disabilities. Although targeted through interventions by community organizations, healthcare professionals and municipalities, little is known about the needs of older adults to participate socially, especially in rural areas. This study thus aimed to identify and prioritize the social participation needs of older adults living in a rural regional county municipality.

**Methods:**

A participatory action research was conducted in a rural regional county municipality (RCM) in Quebec, Canada, with a convenience sample of 139 stakeholders, including older adults, caregivers, healthcare and community organization managers, healthcare and community organization workers, community partners and key informants.

**Results:**

Facilitators and barriers to social participation are related to personal factors (e.g., health, interests, motivation), the social environment (e.g., availability of assistance or volunteers) and the physical environment (e.g., distance to resources, recreational facilities and social partners). Nine older adults’ needs emerged and were prioritized as follows: 1) having access to and being informed about transportation options, 2) being informed about available activities and services, 3) having access to activities, including volunteering opportunities, suited to their interests, schedule, cost, language and health condition, 4) being accompanied to activities, 5) having access to meeting places near home and adapted to their health condition, and 6–9 (no preferred order) being reached when isolated, being personally invited and welcomed to activities, having a social support network, and being valued and recognized. Differences emerged when prioritizing needs of older adults with disabilities (greater need for assistance, accessibility and adapted activities) and older adults living in a rural area (greater need for transportation).

**Conclusions:**

To promote active participation in the community, the social participation needs of older women and men living in rural areas must be addressed, especially in regard to transportation, information, adapted activities, assistance and accessibility. The first part of this action research will be followed by community selection and implementation of initiatives designed to ultimately foster their social participation.

## Background

### Healthy aging: one of the most important challenges facing individuals and societies, including rural communities

While longer life expectancy increases the number of people aged 65 and older around the world, longevity is not necessarily without disabilities [1]. About a quarter of older adults live in rural communities [2] and face specific challenges, such as restricted access to some health and social services, and limited housing and transportation options [3]. Moreover, approximately half of older adults will present disabilities at some point [2]. Since a common desire of older adults is healthy aging [1], interventions on determinants of health such as social participation are essential.

### Social participation: among solutions to foster healthy aging

Social participation is defined as a person’s involvement in activities that provide interaction with others in the community [4]. In meeting their basic socialization and self-actualization needs, it is critical for retired older adults to develop abilities and make life meaningful, promote health, and prevent disabilities [5]. Even in industrialized countries, many older adults do not have equitable opportunities to achieve full social participation due to, for example, inequitable access to activities and information. In Canada, approximately half of older adults experience restricted social participation [2]. Diminished social participation is a critical element affecting older adults’ health [1] resulting in a 29% greater risk of mortality [6]. However, it is possible to intervene to maintain or increase social participation by optimizing a person’s abilities and environment [7], e.g., improved mobility, or providing an environment that promotes interaction. Interventions targeting the social participation of older adults in rural communities are currently delivered by community organizations, healthcare professionals and municipalities across Canada. However, they take little advantage of older adults’ personal and environmental resources [8] and reach only a limited number of people [9], which increases health inequities [10].

To foster social participation, it is important to know more about the needs of older adults. One multiple case study found that a majority of urban older adults with disabilities had needs that were unmet, especially for social activities, including responsibilities, interpersonal relationships, community life and leisure, and for some daily activities such as fitness, as well as housing and mobility outside the home [11]. Older adults and their caregivers often have limited knowledge about interventions available in the community and limited ability to use them. Even healthcare providers report having limited knowledge of interventions available in the community and limited time to evaluate and implement interventions to meet older adults’ needs, including supporting them and their caregivers in taking steps to use community services [11]. To improve and maintain older adults’ social participation, needs should be assessed more comprehensively, including social activities. More support should also be provided regarding the use of resources, and partnerships with community organizations should be optimized. Interventions fostering age-friendly environments should also be targeted.

### Environmental factors: strengths and challenges in social participation in rural communities

As interventions on environmental factors may have a greater impact on social participation than those targeting individual factors [12], it is important to identify environmental facilitators for, or barriers to, doing social activities in rural communities [7]. However, few social participation studies have been carried out with rural older adults [13] or have compared those living in metropolitan, urban and rural areas. According to three quantitative studies, the social participation of older Canadians was found to be similar in metropolitan, urban and rural areas [14–16] but was associated with different environmental factors [15]. In all areas, greater proximity or accessibility to resources and having a driver’s license were positively associated with social participation. However, public transportation use and the quality of the social network were associated only with the social participation of older adults living in metropolitan areas while the presence of children living in the neighborhood and more years spent living in the current dwelling were correlates of social participation identified in rural areas [15]. The social participation of aging adults in metropolises was higher than in medium and large urban centers, but similar to rural and small centers [16]. Barriers show that rural and metropolitan respondents were most likely to report being too busy to participate more, and less likely to report limitations due to health conditions. However, one respondent out of ten in rural areas, i.e., twice the proportion found in metropolises, reported that certain activities were not available [16]. Finally, results support the importance of various physical factors (e.g., user-friendliness of the walking environment) as well as social factors (e.g., support from family and friends) that affect the social participation of older adults [15]. Despite widespread acceptance of the role of the environment in social participation, a comprehensive portrait of the needs of older adults living in rural areas to participate socially is still lacking. Greater understanding could help community organizations, healthcare professionals and municipalities to craft interventions to enhance the social participation of older adults living in rural communities. This study thus aimed to identify and prioritize the social participation needs of older adults living in a rural regional county municipality (RCM).

## Methods

This study was embedded in a larger research program aimed at implementing initiatives to foster the social participation of older adults in rural communities. It used a participatory action research (PAR) design, i.e., an iterative process focusing on the development, validation and implementation of an action [17]. This design was chosen to explore the factors influencing social participation and facilitate the identification of actions and means adapted to the context, a process that is more likely to be effective in changing practices [18]. The present study focused on the first two steps of the process proposed by Dolbec and Clément [17], namely perceiving a problem or concern and clarifying the situation through the gathering of information, in this case identifying and prioritizing the social participation needs of older adults living in a rural area.

The study was conducted in a 1350 km^2^ rural RCM with 19,000 inhabitants, two small cities and ten towns (range: 100–9200 inhabitants) located in the Eastern Townships of Quebec, Canada. An intermediate level of government, a RCM is a group of municipalities in the same territory that cooperate on planning and on the development and implementation of regional policies that increase population well-being and reduce inequities [19]. The RCM where the study was conducted is characterized by the presence of rural and small urban areas, and both French- (about 90%) and English-speaking communities. Among the 3200 older inhabitants, about 2000 are in a vulnerable situation, i.e., are not physically active, have unhealthy life styles, are socially isolated, or are at risk of being abused or neglected [20]. The stakeholders (community organizers, seniors’ consensus committee and community partners) initiated this study and set up a governance committee to guide and support it (e.g., recruit participants, validate data analysis). This mobilization of stakeholders is a key component of successful and lasting community development. The research ethics committee of the *CIUSSS de l’Estrie-CHUS* approved this study (#2015–464). To protect identity and privacy, the participants’ names were replaced by a code. Consent forms were kept in a binder in a locked desk, and the transcripts were stored on the Research Center’s secure computer network.

### Participants

To reach and mobilize a significant part of the population, as well as foster deep exploration and data saturation, a total of 139 participants/experts were involved in the study: 61 adults aged 65 or older, 18 caregivers of older adults, nine healthcare and community organization managers, ten healthcare and community organization employees, ten community partners (e.g., pharmacist, rural and cultural development officer) and 31 key informants from the RCM. The key informants were all members of the seniors’ consensus committee, who represented of the region’s older adults and involved in improving and maintaining their quality of life in the RCM as well as their development in society. At the beginning of the study, no personal or professional connections existed between the research team and the participants and partners. Participants were involved in needs identification (*n* = 86), prioritization (*n* = 113) or both (*n* = 60). While only 26 of the participants who helped to identify needs were not involved in prioritizing them, none dropped out of the focus groups. The older adults and caregivers lived in the RCM, had preserved cognitive functions based on interviewer judgment, and could communicate orally. Managers, employees, partners and key informants worked with the aging population in the RCM. Participants were recruited using a convenience strategy, including advertisements in local newspapers and public places, and word of mouth. To ensure diversity, recruitment of older adults targeted both women and men, with and without disabilities, who lived in urban and rural areas, and spoke French or English.

### Data collection and tools

To identify needs, 16 focus groups and two individual interviews lasting approximately 90 min were conducted between February and June 2015. Eight focus groups involved older adults, three with caregivers, and one with every other category of participants, i.e., managers of healthcare services, providers of healthcare services, community organization managers, community organization employees, and partners. Key informants from the RCM participated in these groups according to their profile. Among the eight focus groups with older adults, one exclusively involved older adults with disabilities, another included English-speaking older adults, and a third comprised older men. This strategy was designed to target different cultural and living conditions that might influence older adults’ perceptions and needs regarding social participation. In addition, homogeneous groups can facilitate openness among the participants during discussions. The focus groups and interviews were conducted by four female interviewers of different ages (30 to 40 years old), with different academic backgrounds (occupational therapy, gerontology, sociology, health promotion, special education and speech therapy) and with different roles in the research team (one academic researcher (ML) and three experienced research assistants, including SR). Focus groups and interviews were held in a library, a volunteer center and a health center. Participants were warmly welcomed by the research team and offered snacks and beverages. They were told that there were no “correct answers” and comments would be kept confidential. A semi-structured guide was pretested and used to stimulate discussion about facilitators and barriers to social participation to help with identifying needs. The guide included open-ended questions such as: “What helps you to participate in your activities?”, “What prevents you from doing your activities?”, and “Overall, what is necessary for you to take part in activities in your community?” to identify needs. The semi-structured guide was validated by the seniors’ consensus committee (for example, an effort was made not to stigmatize the isolation of older adults). Interviewers avoided asking questions that could influence participants’ responses and, when necessary, they reformulated questions or responses to ensure or verify comprehension, respectively. The research team also discussed preconceived ideas and hypotheses. The data saturation point was reached, i.e., data collection was considered complete when the material collected no longer produced new ideas. All groups and interviews were digitally audiotaped and transcribed for further analysis. After each group and interview, a summary prepared by an observer was presented to the participants. Most of the time, this was done verbally right after the meeting or by mail, followed by a phone call, to validate the interpretation of the main content of the exchanges. This validation strategy ensured that the interpretation of the discussions did not overlook individual accounts but did encourage/advocate the co-construction of knowledge.

To prioritize needs, 12 forums, i.e., groups of experts (study participants) who discussed and adopted proposals by consensus, lasting approximately 60 min, were held between November 2015 and January 2016. During these forums, three steps were followed. First, identified needs were explained, orally and in written form (handouts), to participants in a random but consistent order. This explanation of the previously identified needs also validated earlier analyses as participants indicated their agreement with the needs presented. Second, participants individually rated the three most important needs for the social participation of older adults in the RCM. After doing this rating for older adults in the RCM in general, participants were asked to repeat this procedure twice, specifically for groups of older adults with disabilities and those living in a rural area. Third, during each forum, individual prioritizations were summed and transposed onto paper boards to examine trends and initiate discussion. A semi-structured guide was used and included open-ended questions such as: “What do you think about the results concerning older adults in general?” and “In your opinion, which results adequately reflect the social participation needs of older adults in the [name of the main city] area? Explain.” Five stakeholders, i.e., one community organization manager and four partners, could not attend the forums so they completed the prioritization grids electronically and sent in their responses by email. All forums were digitally audiotaped and summarized to enrich interpretation of the prioritization process. A self-administered questionnaire was used to collect the usual sociodemographic and clinical data.

### Data analysis

To describe their context, the participants’ sociodemographic characteristics were described by means, standard deviations, median and interquartile interval, or frequencies and percentages according to the type of variable (continuous or categorical, respectively). Data related to number of diseases, marital status, relationship with the older adult, living arrangement, main language or feeling depressed were collected for older adults and caregivers (but not for healthcare and community employees, managers, community partners or key informants). To identify factors associated with social participation and needs, the groups and interviews were analyzed for thematic content as described by Miles, Huberman and Saldana [21]. To provide information about their recurrence, overlap, combination, complementarity and discrepancies [22], themes and content emerging from the qualitative data analysis were also quantified. Themes were classified according to an extraction grid based on the interview guide developed from the Human Development Model–Disability Creation Process (HDM-DCP), an anthropological model of human development and disability. The HDM-DCP illustrates interactions between intrinsic personal factors (e.g., age, gender, abilities), extrinsic physical and social environmental factors (e.g., distance to resources, family), and participation. Consistent with the constructionist perspective, themes that emerged were only later organized and defined using the coding guide based on the HDM-DCP. Two research assistants coded the data and one third was co-coded by other members of the team. In line with PAR, results were iteratively presented to, discussed with and enriched by the governance committee to ensure that needs and prioritization reflected real-world experiences. Analyses were performed using NVivo (version 10.0) software. Prioritization of needs was calculated by adding the scores assigned by each participant, who identified the most important (three points), second most important (two points) and third most important need (one point). The sum was performed for each of the three populations in the following order: older adults in the RCM in general, those with disabilities, and those living in a rural area. Frequencies and the total score were calculated for each need in each population. Finally, needs were combined for the overall situation (all three populations considered together).

## Results

Older participants were mainly women aged 65–92, French-speaking, with more than seven years of education, living with their spouse, and residing in the RCM for over 15 years (Table 1). Three out of five older participants reported at least one disease and about two out of five felt depressed. Caregivers were women aged 43–86, with the majority having a high school diploma (Table 1). Half the caregivers cared for a parent and had done so for more than seven years. Most lived with a spouse or family member, reported no disease and did not feel depressed (Table 1). Healthcare and community organization managers and employees, partners and key informants were mostly women and the majority had at least seven years of experience within the RCM (Table 1). From the interviews and focus groups involving all these participants and from discussions with the governance committee, important factors for the social participation of older adults, i.e., facilitators and barriers, were identified, followed by social participation needs. Lastly, participants prioritized these needs.

**Table 1:** Participants’ characteristics

	Older adults (***n*** = 61)	Caregivers (***n*** = 18)	Healthcare providers and community organization employees (***n*** = 10)	Healthcare and community organization managers (***n*** = 9)	Community partners (***n*** = 10)	Key informants (***n*** = 31)
Continuous variable	Mean (S.D.^a^)	Median (I.Q.R.^b^)^c^	Median (I.Q.R.)	Median (I.Q.R.)	Median (I.Q.R.)	Mean (S.D.)
Age [years]	78.0 (12.0)	64.0 (9.3)	39.5 (22.8)	53.0 (18.5)	38.5 (22.0)	50.0 (28.5)
Categorical variables	n (%)	n (%)	n (%)	n (%)	n (%)	n (%)
Gender [women]	41 (67.2)	18 (100.0)	8 (80.0)	7 (77.8)	8 (80.0)	24 (77.4)
Education						
None	2 (3.3)	0 (0)	0 (0)	0 (0)	0 (0)	0 (0)
Elementary school	12 (19.7)	0 (0)	0 (0)	0 (0)	0 (0)	0 (0)
High school	27 (44.3)	11 (61.1)	0 (0)	0 (0)	0 (0)	4 (12.9)
College/certificate/ professional diploma	17 (27.9)	4 (22.2)	4 (40.0)	1 (11.1)	4 (40.0)	10 (32.3)
Bachelor’s degree	2 (3.3)	2 (11.1)	4 (40.0)	4 (44.4)	3 (30.0)	3 (9.7)
Master’s/doctoral degree	1 (1.6)	1 (5.6)	2 (20.0)	4 (44.4)	3 (30.0)	12 (38.7)
*Missing*	*0 (0)*	*0 (0)*	*0 (0)*	*0 (0)*	*0 (0)*	*2 (6.5)*
Number of years…	… living in the RCM	... as a caregiver	… of experience within the RCM	… of experience within the RCM	… of experience within the RCM	… of experience within the RCM
< 1	1 (1.6)	0 (0)	2 (20.0)	0 (0)	1 (10.0)	3 (9.7)
1 to 4	3 (4.9)	4 (22.2)	1 (10.0)	0 (0)	4 (40.0)	4 (12.9)
5 to 14	13 (21.3)	9 (50.0)	3 (30.0)	6 (66.7)	4 (40.0)	8 (25.8)
≥ 15	44 (72.1)	3 (16.7)	4 (40.0)	3 (33.3)	1 (10.0)	13 (41.9)
*Missing*	*0 (0)*	*2 (11.1)*	*0 (0)*	*0 (0)*	*0 (0)*	*3 (9.7)*
Number of diseases						
0	25 (41.0)	11 (61.1)				
1	19 (31.1)	4 (22.2)				
2	12 (19.7)	2 (11.1)				
3	5 (8.2)	1 (5.6)				
Marital status						
Married/Common-law	30 (49.2)	10 (55.6)				
Widowed	26 (42.6)	4 (22.2)				
Single (never married)	3 (4.9)	3 (16.7)				
Divorced/Separated	2 (3.3)	1 (5.6)				
Relationship [parent: yes]		9 (50.0)				
*Missing*		*2 (11.2)*				
Living arrangement						
Alone	28 (45.9)	7 (38.9)				
Lives with partner/spouse	31 (50.8)	8 (44.4)				
Lives with family member	2 (3.3)	3 (16.7)				
Main language [French]	55 (90.2)	17 (94.4)				
Feel depressed [yes]	11 (18.0)	2 (11.1)				
*Missing*	*2 (3.3)*	*0 (0)*				

^a^ *S.D.* Standard deviation

^b^ *I.Q.R*. Interquartile range

^c^ For samples smaller than *n* = 30, medians and interquartile ranges were calculated

### Factors reported as important for social participation

All factors that influenced social participation were reported as potential facilitators or barriers, depending on each participant’s experience (Table 2). For example, a family can be supportive, visit often and facilitate the social participation of one older adult, while a family living far away, unavailable to help, might be a barrier for another. However, five factors, three personal and two from the social environment, were principally facilitators, while one personal factor was mainly a barrier (Table 2).

**Table 2:** Factors reported by participants as important for social participation in the RCM

**Personal factors**	
Interest in social activities and participation	
Desire to stay active and socialize (+)^a^	
Habit of being socially active (+)	
Desire to be involved in the community and volunteer (+)	
Motivation and initiative (−)	
Health	
**Physical environment**	
Availability and accessibility of infrastructures and meeting places	
Distance to resources, recreational facilities and social partners	
**Social environment**	
Availability and organization of transportation	
Availability of assistance (+)	
Invitation to activities, solicitation and recruitment process	
Availability of activities devoted to older adults and suited to their needs	
Identification of interests and needs of older adults in the area (+)	
Support and presence of family and friends	
Availability of volunteers	
Responsibilities and support related to caregiving role	
Family habits of meeting, phoning and visiting the older adult	
Sustainability of activities	
Availability of information concerning activities and resources	
Attitudes toward older adults	

^a^ Although all are potentially both barriers and facilitators, depending on experience, some factors were reported as mostly fostering (+) or impeding (−) social participation

Among personal factors, desire, motivation, interests and habits with respect to being socially active were important for social participation (Table 2), as highlighted by participants from one group of older adults (O): ‘*My husband and I have been involved with many organizations since we were young [ …]. We did a lot of volunteering and we still do [ …]. When there are volunteer dinners, we go! We don’t miss anything!*’ (O6: number identifies the discussion from which the quote was taken). The desire of older adults to be involved in the community and volunteer was also an important factor (Table 2), as reported in another group of older adults: ‘*I’d like to help someone who is unable to go out.*’ (O8). Finally, the health of older adults was also influential (Table 2), as described by one group of workers from healthcare and community organizations: ‘*[My mother] is in great shape. She is involved in a lot of things.*’ (Workers, W1).

Among physical environmental factors, the availability and accessibility of infrastructures and meeting places were important for social participation (Table 2): ‘*Not all towns and villages have meeting rooms.*’ (O2). Distance to resources, recreational facilities and social partners were also reported as factors that influenced social participation: ‘*There’s one thing with paratransit; it’s easier for older adults in the city to use [compared to those living in rural areas]. They’re closer to it.*’ (Community partner, C2).

Finally, many social environmental factors such as the availability and organization of various resources such as transportation, assistance, adapted activities, volunteers and information were reported as important for the social participation of older adults living in the rural RCM (Table 2): ‘*We know, transportation is a limitation here [ … ] Since it is limited here, older adults cannot take part in certain activities.*’ (Healthcare and community organization managers, M3)*.* The support, presence and habits of family and friends in interacting were also important, as noted by these older adults with disabilities: ‘*My husband pushes me [in the wheelchair]. Also, when we visit our children, one of my sons brings me up to his house. Our children are very helpful.*’ (O4-Disability).

Factors that influence social participation were often reported as being interrelated. For example, when health was not an issue, organizing transportation was easier when older adults still drove their own car. However, when older adults who lived far from the main town experienced declining health, the availability of volunteers to assist and transport them became important. Discussing facilitators and barriers in focus groups beforehand generated a better understanding of the overall situation and helped the process of identifying needs.

### Social participation needs of older adults

Nine needs emerged from the discussion concerning factors influencing social participation (Table 3). One third of those needs focuses on adapting activities or the physical environment (accessibility, adapted activities and transportation), another third mainly targets the initiation of activities (information, identification and personalized approach), while the remaining needs relate to the social environment (assistance, support network and recognition).

**Table 3:** Prioritization of older adults’ needs by population

	In general (***n*** = 110)	With disabilities (***n*** = 107)	Living in rural areas (***n*** = 106)
**Needs related to adaptation of activities or physical environment**	n (%)	n (%)	n (%)
**Accessibility** (Having access to meeting places near home and adapted to their health condition)	**59; R5**^**a**^	**97; R3**	**57; R5**
Priority 1	9 (8.2)	14 (13.1)	7 (6.6)
Priority 2	4 (7.3)	18 (16.8)	11 (10.4)
Priority 3	24 (21.8)	19 (17.8)	14 (13.2)
**Adapted activities** (Having access to activities, including volunteering opportunities, suited to their interests, schedule, cost, language and health condition)	**114; R2**	**114; R2**	**49; R6**
Priority 1	17 (15.5)	21 (19.6)	7 (6.6)
Priority 2	23 (20.9)	21 (19.6)	10 (9.4)
Priority 3	17 (15.5)	9 (8.4)	8 (7.5)
**Transportation** (Having access to and being informed about transportation options)	**46; R8**	**91; R4**	**185; R1**
Priority 1	4 (3.6)	10 (9.3)	36 (34.0)
Priority 2	12 (10.9)	21 (19.6)	31 (29.2)
Priority 3	10 (9.1)	19 (17.8)	15 (14.2)
**Needs related to initiating activities**	n (%)	n (%)	n (%)
**Information** (Being informed about available activities and services)	**166; R1**^**a**^	**40; R6**	**120; R2**
Priority 1	43 (39.1)	7 (6.5)	28 (26.4)
Priority 2	17 (15.5)	5 (4.7)	12 (11.3)
Priority 3	3 (2.7)	9 (8.4)	12 (11.3)
**Identification** (Being reached when isolated)	**63; R4**	**32; R8**	**59; R4**
Priority 1	13 (11.8)	5 (4.7)	12 (11.3)
Priority 2	9 (8.2)	5 (4.7)	13 (4.7)
Priority 3	6 (5.5)	7 (6.5)	5 (12.3)
**Personalized approach** (Being personally invited and welcomed to activities)	**50; R7**	**36; R7**	**47; R8**
Priority 1	4 (3.6)	3 (2.8)	3 (2.8)
Priority 2	13 (11.8)	10 (9.3)	12 (11.3)
Priority 3	12 (10.9)	7 (6.5)	14 (13.2)
**Needs related to social environment**	n (%)	n (%)	n (%)
**Assistance** (Being accompanied to activities)	**52; R6**^**a**^	**139; R1**	**49; R7**
Priority 1	4 (3.6)	24 (22.4)	4 (3.8)
Priority 2	14 (12.7)	25 (23.4)	11 (10.4)
Priority 3	12 (10.9)	19 (15.9)	15 (14.2)
**Support network** (Having a social support network)	**75; R3**	**74; R5**	**60; R3**
Priority 1	14 (12.7)	21 (19.6)	8 (7.5)
Priority 2	10 (9.1)	2 (1.9)	12 (11.3)
Priority 3	13 (11.8)	7 (6.5)	12 (11.3)
**Recognition** (Being valued and recognized)	**27; R9**	**19; R9**	**10; R9**
Priority 1	2 (1.8)	2 (1.9)	1 (0.9)
Priority 2	4 (3.6)	0 (0.0)	2 (1.9)
Priority 3	13 (11.8)	13 (12.1)	3 (2.8)

^a^ Score; Rank (R) representing the order of the total scores summing priorities given by each participant who ranked needs as first (3 points), second (2 points) or third (1 point) most important need. No points were assigned to other needs not ranked as first, second or third

First, needs were related to the adaptation of activities or physical environment, such as having access to meeting places, as mentioned by the group of English-speaking older adults: ‘*[We need] a center where we could go, you know, a place we could go to.*’ (O7-English). Places for socializing and doing activities include community halls, local businesses and churches. These places must also be adapted to health conditions, with access ramps and suitable maintenance, including snow removal. Having access to activities adapted to their needs is important for older adults: ‘*We need to find activities, either walking or … I don’t know … cycling. However, 70-year-olds won’t be cycling. We need to try to vary the activities offered.*’ (O2) or ‘*Just an afternoon of getting together and we could do whatever people bring with them, it’s still a social exchange.*’ (O7). Activities should be interesting for older adults, tailored to their schedule, offered at a reasonable cost, in their main language and suited to their health conditions. The last need identified involved information about access to transportation options: ‘*We will need to have transportation, which can be carpooling, partnering, etc.*’ (Healthcare and community organization managers, M2) and ‘*People should know transportation services that can go and get people in the villages, take them to the community center or any other activity.*’ (O8). All kinds of transportation were discussed, including public transportation, assisted transportation and individual transportation [own car]. As for activities, transportation must be suited to the schedules, budget and health conditions of older adults, including those with mobility issues.

Second, regarding initiating activities, older adults need to be better informed about social opportunities, as illustrated by this quote from one group of healthcare and community organization workers: ‘*We should find ways to ensure that information gets to people, that people understand how to access resources, as clearly as possible.*’ (Workers, W2). Such information can reach older adults themselves or anyone interacting with them, for example, their family or healthcare providers. Second, it is necessary to reach out to older adults who are isolated, as reported by one group of managers: ‘*Nowadays, nobody knows “who” is isolated, “who” has needs in the municipality. This information is lacking.*’ (Healthcare and community organization managers, M1) and by one older adult: ‘*It’s not just waiting for the older adults to get moving, it’s getting [information to them] in their homes.*’ (O8). More isolated older adults need to be identified, located and helped when they have less contact with others. Often, older adults also need to be personally invited and welcomed to activities: ‘*It’s not because they don’t want to, but I have the feeling that it would take someone to come pick them up, someone who can motivate them.*’ (Workers, W1). Upon arrival at activities, this personalized approach should include a warm welcome, which could be from family members, relatives, healthcare providers, community organizations, volunteers or other contacts.

Among needs related to the social environment, some older adults need assistance in initiating or taking part in activities in the community: ‘*Some older adults could go [to the activity], but they would need to be accompanied for a while, so they don’t show up alone.*’ (Community partners, C1). Such support could be provided when the older adult does an activity for the first time, or more regularly, to ensure that a routine is established to participate in the activity. Social support from a network is also important to simply socialize, to provide psychological support, or to help with domestic tasks (for example): ‘*There are people who need our help. Just to talk … they are alone. They are just happy when we go and talk to them.*’ (O2). Being valued and recognized was one of the nine needs of older adults, as noted by one group of managers: ‘*We have to value our older adults if we want them to participate socially. They need to feel that they’re important, that they have something to say, and that they’re still helpful.*’ (Healthcare and community organization managers, M4). Such acknowledgement refers to the feeling of making a significant contribution to the community, having status in society, and being respected and appreciated by others.

Specifically, these nine needs identified by all types of participant mainly targeted older adults, not stakeholders, the community or healthcare organizations. Two needs, i.e., ‘being healthy’ and ‘wanting to participate’, were both indirectly considered under the needs ‘having access to activities suited to their needs’ and ‘being personally invited and welcomed to activities’, respectively. As they involved mainly personal factors and might be seen as prerequisites for social participation, these two needs were not directly targeted in the following step.

### Prioritization of needs

**Fig. 1:**
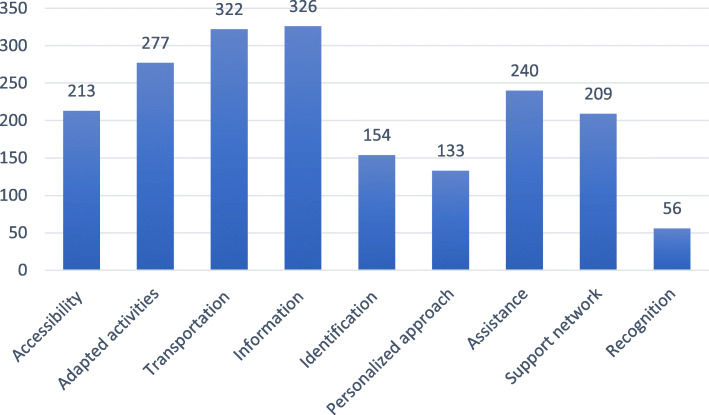
Frequency of overall prioritization of older adults’ needs in a rural regional county municipality

Overall prioritization, i.e., when the perspectives of the three populations were merged, suggested six important needs for older adults in the RCM (Fig. 1; above 200 points), some of which are interrelated. For example, needs for information, assistance and transportation were frequently discussed as being closely related to the social support network that could inform, transport and accompany the older adult. Specific prioritization, i.e., considering each population separately, revealed differences in social participation needs (Table 3). When targeting older adults in general, the main needs were information, adapted activities, support network and identification. For older adults with disabilities, the needs prioritized were assistance, adapted activities, accessibility and transportation. Finally, transportation and information were two central needs of older adults living in a rural RCM.

## Discussion

This participatory action research aimed to identify and prioritize the social participation needs of older adults living in a rural RCM, including both rural and small urban areas. Personal and environmental, but especially social, factors were reported as facilitators and barriers to social participation and guided the identification of nine needs. Six of these needs were prioritized when overall results were considered. The four remaining needs had no preferred order. However, the prioritization of needs differed when done from the perspectives of older adults in general, those with disabilities or those living in rural areas.

Despite recent interest in the development of age-friendly communities, literature about the geography of aging, i.e., understanding the relationships between the physical/social environment and the elderly, is scarce [23], especially literature pertaining to the social participation needs of older adults in rural areas. As mentioned by Menec and colleagues [24], the age-friendliness of communities has received less attention in the context of rural settings compared to urban. The present study therefore sheds new light on the needs of older adults in rural RCMs, including variations in priority needs from a general as well as specific perspectives of older adults with disabilities or those living in rural areas. Since a good understanding of normative conditions may help developers of interventions to better engage older adults who do not conform to the dominant perception of community vision and functioning [25], these distinctions could foster successful implementation of social initiatives with older adults. A good understanding of community functioning is required to shape context-sensitive interventions to counter, for example, social isolation in later life.

Some results of the present study are similar to those of a previous Canadian qualitative study [3]. Through focus groups with older adults and caregivers, factors such as adapted activities, transportation, prevention of isolation, access to outdoor spaces and buildings, and information about activities were found to be related to social participation in age-friendly rural communities. In Canada, all provinces have initiated age-friendly community processes [26], and approximately 800 communities have launched age-friendly initiatives. An age-friendly community encourages active aging by optimizing opportunities for health, participation and security and by adapting its structures and services so they are accessible to, and inclusive of, older people with varying needs and abilities [27]. Similar to the social participation needs pinpointed in the present study, most common projects identified in a consensus conference in the province of Manitoba (Canada) were related to outdoor spaces, buildings, communications and activities (e.g., walking groups, reaching out to isolated older adults) [24]. These projects vary across communities and change over time, suggesting that social participation needs may also vary from one community to the next or over time. Indeed, the ability of rural communities to become age-friendly is influenced by contextual factors such as size, location, demographic composition, ability to secure investments, and leadership [28]. The present study complements this knowledge with evidence of the importance of personalized needs assessments and considering prioritizing needs from several perspectives when planning and implementing initiatives to promote social participation, even within the same rural RCM.

Also consistent with some needs identified in this study, another Canadian study conducted in one urban and three rural age-friendly communities in Manitoba found that to promote health and well-being and facilitate independent living, it is important to ensure that older adults have access to a broad range of community supports, such as the provision of services, counseling, congregate meals, volunteer drivers, and a medical equipment-lending program [29]. For example, transportation links older adults, not only to healthcare services, but also to community life, including local businesses, services and opportunities for social participation. Hence, the absence of affordable, accessible transportation may contribute to social isolation. In addition to transportation, affordability influences many aspects of older adults’ lives, including housing, the social environment, activities and volunteering, community supports, and health services [29].

When considering overall results, transportation and information needs of older adults living in a rural RCM were strongly prioritized by the participants. These results are in line with previous studies which observed that transportation and communication were vital to enhance the social participation of people living in rural areas [24]. Although one cross-sectional quantitative study found that social participation was similar across different types of residential areas in Quebec (Canada), associated area-specific environmental variables were identified [15]. Specifically, in rural areas, greater social participation was associated with greater accessibility to key resources, having a driver’s license, children living in the neighborhood, and more years spent living in the current dwelling. In fact, social participation needs may vary from one community to the next in the same area since rural communities are not all homogeneous [30, 31]. Such diversity requires a multisite approach that considers not only proximity to cities as a means of differentiating between rural places but also the emergence of distinct combinations of demographic, socioeconomic and policy challenges across rural spaces. For example, in the present study, the smallest village had 100 inhabitants and was 60 min from an urban center, while the largest village had 3200 inhabitants and was 15 min from an urban center. Such different realities might explain why transportation and information are more significant issues for populations further from the city, and their need to address these important challenges to promote social participation.

### Strengths and limitations of the study

To our knowledge, this action research is the first to identify and prioritize the social participation needs of older adults living in a rural RCM using a process in which stakeholders played a significant role. In accordance with the guidelines of Raymond and colleagues [9], this action research directly involved older adults, caregivers and community members from many different backgrounds, and therefore provides an inclusive understanding of the needs of older adults in a rural RCM. The study was based on a strong partnership with the community and involved a personalized approach to the experiences and cultures of older adults in the RCM. Moreover, as recommended by Laperrière [32], having several sources of data and participants enabled triangulation, rich information and good internal validity. The limitations included recruitment based on a convenience strategy, where the sample may include more active or healthier older adults, although participants with disabilities, older men and English-speaking older adults were specifically recruited. Older adults with different social participation needs, including men and ethnic minorities, might be underrepresented, as well as people living in rural versus small urban areas. As with other qualitative studies, the findings of this study are time- and context-sensitive and influenced by the researchers. Despite using various strategies (no “correct answers”, confidentiality assured and homogenous groups), the nature of the questions could also have been subject to a social desirability bias and limited the sharing of facilitators, barriers and needs. Lastly, the study involved only one RCM and needs to be reproduced in other areas.

## Conclusion

This action research involved older adults, caregivers and other stakeholders in an effort to identify and prioritize what this older rural population needs in order to be able to participate socially. Personal and environmental, but mostly social, factors were reported as facilitators for, or barriers to, the participation of older adults in the RCM. Specifically, findings underline the importance of adapting activities (e.g., according to interests and abilities), the physical environment (e.g., places for socializing), initiating activities (e.g., information about activities and resources, personalized approach) and the social environment (e.g., assistance, support network). In line with these important factors, six social participation needs were prioritized overall: transportation, information, adapted activities, assistance, accessibility and support network. Prioritization of the needs of older adults with disabilities highlighted that they mostly needed assistance, accessibility, adapted activities and transportation, while those living in a rural area primarily needed transportation and information about activities and services.

To promote the social participation of older adults in a rural RCM, identification of personal and environmental facilitators and barriers, as well as possible variations in the needs prioritized according to specific perspectives, such as older adults with disabilities or living in a rural area, should be considered. The first part of this action research will be followed by community selection and implementation of initiatives to meet older adults’ needs [33, 34]. These initiatives will also be evaluated in terms of older adults’ social participation and health. This type of community mobilization will ultimately reduce the isolation and vulnerability of older adults living in a rural RCM.

## Data Availability

Data are available upon request to corresponding author.

## Abbreviations

HDM-DCP: Development Model–Disability Creation Process
PAR: Participatory action research
RCM: Regional county municipality
